# Neuralgia

**Published:** 1869-08

**Authors:** Chas. G. Edwards


### ARTICLE III.

### *Neuralgia.*

By Chas. G. Edwards, D. D. S.

Neuralgia, a word derived from two Greek words, *neuron*, a nerve, and, *algos*, pain, is a term applied to an affection, characterised by acute, lancinating pains in one or more parts, or organs of the body, the nature of which, being located in the most delicate, highly organized and sensitive tissue of the animal economy, presents an entertaining study to the student of medicine; and is invested with no less interest and importance to the student, who selects Dental Surgery, as a specialty of medicine.

I design confining myself in this paper, more particularly to that form of neuralgia, that comes more directly under the observation of the dental practitioner, and deem no apology necessary for the remarks I shall make upon the disease in general.

The term Neuralgia has been substituted for that of Tic-doloureux, and is employed to designate a pain of a purely nervous character, situated in the nerve substance; and differs in this, from other sensations of a painful character, characterized by vascular congestion, inflammation, &c. The affection is always confined to the nerves of general sensibility, and when one or more of these nerves are the seat of this malady, they themselves are the sufferers; whereas, in inflammation the nerves are the vehicles by which painful sensations are conveyed, or transmitted to the sensorium.

Neuralgic pains may be seated in any organ of the body in which these peculiar nerves, or their branches, are distributed. This morbid condition, however, is most frequently manifested in the superficial or peripheral branches, or ramifications of the nerves; probably from their more exquisite sensibility, together with their greater liability to injury by contact with external irritants. In whatever organ or part of the body neuralgic pains make their apperarance, the expression of suffering or pain is varying in both character and intensity; and the medical vocabulary has been utterly exhausted, and still fails to give a correct and concise expression to the sufferring.

The attacks of neuralgia may recur at intervals of only a few moments, or they may take place daily, every other day, or even longer intervals may occur, regular or irregular, and the pain is sometimes continuous, with increased and exalted fits—some writers describe it as burning, darting, twinging in its momentary duration. It is also attended occasionally with spasmodic contraction, or twitching of the muscles of the affected part.

The pathological condition that seems most favorable to an attack of neuralgia, are numerous; some of the most frequent may be summed up as follows:—A weak and delicate constitution; scrofulous rheumatic, gouty and nervous diathesis. It may also attend functional, or organic diseases of the viscera; of the genital organs, especially the uterus; and it is not unfrequently that these organs make their distresses known through this medium. "It has been more than once in my practice," says Dr. Wood, "the immediate precursor or rather the first obvious sign of Bright's disease of the Kidneys, and sirrhosis or other fatal organic affections." And finally, it may be caused by "Malaria, or Marsh-poison"—a name given to that peculiar condition of the atmosphere, that usually gives rise to intermittent fever, and other paroxysmal diseases, and when from this cause, it generally, probably always, assumes the regular intermittent form, so characteristic of that family of paroxysmal diseases eminating from the same source.

Of the exciting causes of neuralgic pains, I might enumerate a great many that act as local irritants, such as diseased, or dead teeth, tumors, wounds, and quite frequently by spiculae of bone and bony excresences pressing upon a nerve in its course through foramina, bony canals, &c. When forming our diagnosis, we must discriminate between neuralgic pains, and those of an inflammatory character, whether it be dependent upon vascular congestion of the neighboring soft parts, or diseases seated in the sheath of the nerve (its neurilemma) known as neurites.

It is generally, but not always, easy to distinguish these pains from those dependent on inflammation by the absence of pain on pressure. Sometimes, when the malady continues for a long time, the parts may assume an inflammatory character, and by the effusion of serum, may become somewhat swollen; when it has gone so far, without the primary stages being known to the practitioner, the case is frequently truly difficult to diagnose. Here the careful practitioner will be able to detect, in very many cases, the true nature of the disease, by the presence of cutaneous sensibility, and the relief of pain by firm pressure, which will indicate neuralgia. Whereas, in inflammation, there is no tenderness of surface, the pain being constant, and greatly augmented by firm pressure.

Neuralgia Facici, or Facial Neuralgia, is that form of the disease, that comes more directly under the observation of the dental practitioner, and it is probably the most frequent, and common of all the different forms of the malady, and is generally the most severe; often the most excruciating pains being occasioned by apparently the slightest causes. At very many times, the causes are so insignificant as to escape the notice of the sufferer—any sudden noise, or jarring, the slightest touch, and sudden changes of temperature will frequently cause the pain to be manifested.

In this form of the disease the morbid condition is seated in the division of the fifth pair, or trifacial nerve, or some of its branches. And when we remember its organ, its bony canals, the adjacent unyielding tissues, with which it is in contact, as it traverses the hidden paths of the interior of the cranium, we can readily understand how any morbid growths of these tissues, exerting undue pressure upon the nerve, may cause a severe form of neuralgia, to detect the true cause of which, will baffle and defy the untiring energy of the most sagacious practitioner. The disease may extend to all the branches of the fifth pair on one side of the head and face; but more commonly it is confined to one of its principal divisions, of which, the infra-orbital is especially liable to be affected. In many instances it is the situated in temporal and dental, and, not unfrequently, the terminal filaments merely become the seat of intense pain; consequently the malady is occasionally found limited to a patch on the cheek, brow, or temple, from which it rarely ever radiates or shifts.

In facial neuralgia, the causes are various. Many times it appears to be purely of a nervous character,—broken health, debilitating diseases, mental depression, fatigue and exposure to wet and cold, and malarious influences, being among the most common causes. And I have no doubt but that it is frequently occasioned by morbid conditions of the gums, alveolus and teeth, or their lining or investing membranes; by irregular crowded and supernumary teeth; and probably by a variety of other causes.

The pains may come on suddenly, may disappear and return without apparent causes, and may be of all degrees of severity, sometimes moderate, at others almost unendurable. When the attack is sudden, it is usually violent—of a darting, tearing character, sometimes radiating along the trunk or its branches, by which its course may be distinctly traced.

When a case comes under our observation, the symptoms of which are pain in the face or adjacent parts, we should direct our attention, first to the condition of the mouth, particularly the teeth, and if we find one in a carious condition we should examine it carefully—if the pulp be exposed, we may expect to find the pain more or less centralized in the carious one, and we may be quite sure of having found the offending organ. But on the other hand, if we find no diseased condition of the mouth, cheek, &c., such as caries of bone or dentine, or inflammation of the soft parts, we should direct our attention to the general health and condition of the patient, as well as to the character of the pain. If it be characterized by intermission, we should suspect the cause to be malaria, and, if we ascertain that the patient has been subject to intermittent fever, or exposed within the past few months to an atmosphere impregnated with malaria, the evidence would be sufficient to justify our diagnosis. If the paroxysms be not well marked by intermisssions, however, and from the statement of the patient, we learn he has not been residing in a malarious locality, we continue our search to detect the true cause of the suffering; and if we are so fortunate as to detect it, we may apply our remedies with a precision, that will seldom fail to bring a speedy relief.

The treatment of neuralgia is established rather upon the results of experience, than upon any scientific precision in the application of remedies, as both the ultimate and proximal causes of nervous phenomena are obscure.

Some causes of neuralgia have been detected, and are well understood, and when it is possible to detect one of these known causes, the remedy is specific, and may be applied with hope of speedy relief. But, when we are not so fortunate as to detect the cause, our remedies are applied in an empirical way, often indeed at random, and our practice degenerates into a series of experiments. At length we strike upon the remedy by accident, or the case yields to the benificence of nature. In this case the last remedy tried is apt to obtain the credit of subduing the disease; hence has arisen a host of vaunted remedies of very opposite nature, and diverse effects upon the system.

One of these known causes of neuralgia, is, as I have said before, malarious poison, and when arising from this cause, can therefore usually, but not always, be detected, and then the remedy belongs to that class of medicines vaguely termed anti-periodics, of which there are a great many of secondary and inferior powers. But the principal are quinine and arsenic, which are prescribed in various doses and combinations to suit the views of the practitioner.

But the power of these remedies is not strictly limited to the cases of malarious origin. Arsenic has been found useful in many other cases, and especially in hemicrania, or where the pain is confined to one side of the head; why this is so is not known.

Quinine also acts beneficially upon many cases where debility is a marked symptom—probably by virtue of its tonic powers. In such cases, it should be combined with some preparation of iron—a combination which will afford prompt relief in very many instances.

When the neuralgia is of an inflammatory origin, such remedies would be productive of harm. Frequently when quinine, iron, arsenic, &c., have been prescribed without effect, if a dose of calomel be given at night, and the ineffectual remedy resumed the next morning, prompt relief will be obtained.

When neuralgia is inflammatory, it is best combatted by the antiphlogistic remedies and anodynes. The disease, when depending upon pressure occasioned by morbid growths, such as I have before mentioned, must be relieved by removal of the morbid growth, or if that be impossible, section of the nerve is the only means of relief. All other remedies will prove unavailing for more than temporary relief, and even section many times fails to give permanent relief, and excruciationg agony will make life a burden, and death will be hailed as a happy relief by the unfortunate sufferer.

A vast variety of liniments and external applications, are recommended in the treatment of this malady, and are often efficacious for temporary, and even permanent relief. But in none of them can it be supposed that any special virtue resides; they all act upon one and the same principle— that of counter-irritation or revulsion, which apparently consists in making a new impression upon the part— upon which the morbid action subsides. Some of these local applications, however, contain anodyne remedies, which are to some extent absorbed by the surface to which they are applied, and act by destroying or impairing temporarily the sensibility of the part. In cases of pure neuralgia which occur in the pale and feeble, we must rely upon those medicines that tend to restore the general health and strength of the patient; these we find in the mineral tonics, of which the salts of iron are the best. The precipitated sub-carbonate of iron, in doses of five to thirty grains is a good preparation; but probably we find the sulphate, or impalpable iron powder the best form in which to administer these remedies—dose one to two grains, two or three times a day. By continuing this treatment for several weeks, the health of the patient will generally improve; and when such is the fortunate result of our treatment, we may confidently expect permanent relief. Whilst we are treating the case with tonics, it may be necessary to paliate the disease by the administration of anodynes, or narcotics, of which opium, conium and hyoscyamus are the most in use. These may be given internally, endermically, or by the more recent method of injection by hypodermic syringe; and they rarely fail to give speedy relief, their action being sometimes instantaneously manifested.

I have mentioned, as one of the causes of neuralgia, morbid conditions of the mouth, teeth, &c. These may cause a very distressing form of the malady, which may even be felt at a distant part.

If it be a tooth, it will generally, but not always, be found tender on pressure, or percussion. If the organ cannot be restored to health, or even if other indications call for its removal, by doing so, will generally be sufficient for a cure. In severe facial neuralgia the mouth ought to be examined, and if we find any dead teeth or fangs they should be extracted without delay, and if any other be found in a state of decay that can be saved by filling, this operation should be performed.

There has recently been brought to the notice of the medical profession, a preparation which is highly recommended in the treatment of neuralgia, by gentlemen whose scientific attainments shonld certainly claim our attention, if not justify us in its use. The name given to this remedy is Narcein. It is one of the active principles of opium, and resembles morphia, not only in the fact that they are derived from the same base, but also in its action upon the nervous system as a sedative and hypnotic. It is claimed for this preparation that it acts beneficially in cases where morphia is either not tolerated from the begining, or where it has lost its effect by long use; it also acts more promptly than morphia. "It produces sleep, which is soft, tranquil, uninterrupted, and followed by a quiet waking," without the unpleasant effects that sometimes ensue from the use of morphia. These are the general principles of the treatment of neuralgia, and by attention to them, most cases may be, more or less, speedily relieved. But occasionally, every expedient will fail, and the disease will continue to torment our patient in spite of our efforts.

When we witness the many wonderful discoveries and improvements in the various departments of art and science, with which the present century teems; when we see the might genius and intellect, which is being directed to the improvement of the condition and the relief of suffering humanity, we are justified in the belief that this hitherto terrible and but partially understood disease, will yet find its panacea in the investigation of science, and will readily yield to the treatment of the skillful.

